# Effect of Prior Transurethral Prostate Resection (TURP) or Laser Enucleation (ThuLEP) on Radiotherapy-Induced Toxicity and Quality of Life in Prostate Cancer Patients Undergoing Definitive Radiotherapy

**DOI:** 10.3390/cancers16193403

**Published:** 2024-10-06

**Authors:** David Rene Steike, Fabian Martin Troschel, Julian Roers, Jan Jakob Siats, Christopher Kittel, Niklas Benedikt Pepper, Stefan Gravemeyer, Philipp Papavassilis, Andres Jan Schrader, Hans Theodor Eich, Sergiu Scobioala

**Affiliations:** 1 Department of Radiooncology, University Hospital Muenster, 48149 Muenster, Germanysergiu.scobioala@ukmuenster.de (S.S.); 2 Department of Urology, University Hospital Muenster, 48149 Muenster, Germany

**Keywords:** prostate cancer, radiotherapy of the prostate, radio-oncology, transurethral resection of the prostate, TURP, thulium laser enucleation of the prostate, ThuLEP, IPSS, quality of life, IMRT

## Abstract

**Simple Summary:**

In the radio-oncological treatment of patients with prostate cancer, there are significant differences in therapy tolerance due to risk factors, previous illnesses, and pre-treatment of the prostate. Patients with previous prostate surgery (TURP or ThuLEP) are a significant risk group in prostate cancer treatment and differ from non-operated patients in terms of quality of life. Patients who underwent transurethral prostate resection (TURP) exhibited greater overall quality of life impairment immediately after radiotherapy compared to those who had transurethral laser enucleation (ThuLEP) or no surgery. Operation time and radiation technique are important factors regarding therapy tolerance.

**Abstract:**

In our study, the post-radiotherapy quality of life of prostate cancer patients who previously underwent transurethral resection of the prostate (TURP) is compared to those who had thulium laser enucleation of the prostate (ThuLEP) and those who had no prior surgery. It also aims to identify and assess risk factors affecting therapy tolerance in this patient group. We analyzed 132 patients with localized prostate cancer treated with definitive radiotherapy (RT), including 23 who had prior TURP and 19 who previously underwent ThuLEP. A total of 62% of patients underwent irradiation within 12 months after surgery. We included only patients treated with radiotherapy using the IMRT technique. Changes in patient-reported urinary toxicity were evaluated using the International Prostate Syndrome Score (IPSS) and the quality of life index of the World Health Organization (QoL/WHO-PSS) over a three-year post-radiotherapy period. Patients with prior TURP experienced significant deterioration in QoL and IPSS immediately after irradiation (*p* < 0.001), whereas those without previous surgery showed both less significant differences in IPSS and QoL scores. In conclusion, patients with previous TURP/ThuLEP differ from those without previous surgery in urinary quality of life and acute and chronic urinary symptom profiles after RT. The surgical technique (ThuLEP vs. TURP) and the time interval to irradiation are crucial factors affecting RT tolerance in acute and late settings. The previously operated patient group reported a significantly longer period of increased symptom burden.

## 1. Introduction

Patients with symptomatic benign prostate hyperplasia (BPH) are at risk of sequentially developing invasive prostate cancer [1]. Prior studies indicate that previous BPH-directed subtotal prostate resection may limit the urinary quality of life after subsequent definitive external beam radiotherapy (EBRT) for prostate cancer [2,3,4], possibly due to the induction of periurethral fibrosis.

Transurethral resection of the prostate (TURP) and thulium laser enucleation of the prostate (ThuLEP) are the two main options for subtotal surgery for BPH. Some data indicate that ThuLEP is associated with less morbidity in comparison with TURP [5,6].

In patients with a history of TURP, subsequent RT can exacerbate pre-existing urinary symptoms. This exacerbation can lead to increased severity of urinary incontinence and obstructive symptoms, often due to urethral stricture [7,8,9,10]. However, other studies have not shown any correlation between previous TURP and increased urinary toxicity after EBRT [11,12]. This inconsistency between studies may be associated with the different specific toxicity scoring systems utilized. Conversely, there are currently no studies evaluating the impact of ThuLEP on urinary function when RT is delivered after the procedure. In addition, intestinal toxicities in patients receiving EBRT after prior TURP or ThuLEP were not evaluated in detail in previous trials.

In this study, we analyzed urinary and rectal toxicity profiles in both acute and late settings and assessed quality of life (QoL) outcomes following modern, intensity-modulated EBRT. We compared these profiles between patients with a prior history of TURP or ThuLEP and those without surgical intervention.

## 2. Materials and Methods

### 2.1. Study Design and Patient Characteristics

Patients ≥50 years of age with localized prostate cancer with and without lymph node involvement were analyzed while patients with relevant comorbidities (≥ECOG 3) and with extensive prior treatment, e.g., prior radiochemotherapy, were excluded. Only patients who received radiation treatment using the IMRT technique were included to analyze a homogeneous irradiation technique. Exclusion and inclusion criteria are detailed in Supplementary Table S1.

A total of 132 patients with localized prostate cancer between 2012 and 2021 were retrospectively analyzed. Three groups of patients were compared: 19 underwent ThuLEP before radiotherapy, 23 underwent TURP, and 90 had no previous surgery. ThuLEP and TURP procedures were performed for benign prostatic hyperplasia. We also collected relevant factors such as androgen deprivation therapy (ADT), age, T stage, Prostate Imaging Reporting and Data System (PI-RADS), risk stratification of prostate cancer, N stage, prostate-specific antigen (PSA) value, and prostate volume from the medical records.

To analyze the quality of life, the International Prostate Symptom Score (IPSS), the World Health Organization (WHO)-based QoL index, and the Common Terminology Criteria for Adverse Events Genitourinary (CTC-GU) and Gastrointestinal (CTC-GI) scores were measured continuously before (baseline) and immediately after RT as well as 3, 6, 12, 24, and 36 months later. Finally, the three groups were compared with each other. Patients with previous TURP (n = 23) versus patients with previous ThuLEP (n = 19), as well as a combined group (TURP and ThuLEP) (n = 42), and patients without surgery (n = 90) were studied.

### 2.2. Analysis of Quality of Life: IPSS, QoL, CTC-GU, and CTC-GI

The IPSS was used to assess the quality of life and to classify the severity of urinary symptoms from baseline measurement (preRT) up to 3 years post-RT [13,14]. A score ≥8 was defined as moderate, and ≥20 was defined as severe.

To assess patient-reported health-related quality of life (QoL) outcomes following definitive EBRT for prostate cancer, we evaluated the QoL score for genitourinary (GU) toxicities using the Prostate Symptom Score, as introduced by Cockett et al. [15]. The QoL score was used as an expansion to the IPSS measure for the detection of even mild radiation-induced GU side effects that might not be represented within the IPSS-based questioning. Consistent with the IPSS, a high QoL score means greater genitourinary symptomatology.

To supplement the assessment of further gastrointestinal and genitourinary toxicities we included standardized, patient-reported outcomes (CTC-GI and CTC-GU) [16].

### 2.3. Transurethral Resection of the Prostate (TURP) and Thulium Laser Enucleation of the Prostate (ThuLEP)

If non-surgical options fail to achieve adequate treatment of benign prostatic obstruction, different surgical approaches are available: resection, enucleation, vaporization, alternative ablative techniques, and non-ablative techniques [17]. The approach used is based on different factors, mainly prostate volume, patient preferences, and availability [17,18,19]. The most common approaches in Germany today are transurethral resection (TURP) and transurethral laser procedures [19]. Traditionally, TURP is the reference standard for prostate volumes from 30 to 80 cm^3^, while anatomic enucleation, including transurethral laser enucleation, can achieve de-obstruction in even larger prostates [17].

### 2.4. Radiotherapy

RT data were gathered from the database of the institution’s Department of Radiation Oncology at Muenster University Hospital, Germany. RT was applied in a conventionally fractionated manner (CFRT) with 1.8 or 2.0 Gy per fraction up to a cumulative dose of 72.0–80.0 Gy. All patients were planned to undergo intensity-modulated radiotherapy (IMRT) minimizing toxicity by limiting the amount of normal tissue irradiated [20]. A total of 29 patients received a static IMRT using a sliding-window technique. Most patients (n = 77) were treated with a helical dynamic IMRT using tomotherapy (Accuray, Sunnyvale, CA, USA) while n = 26 received a volumetric modulated intensity arc therapy (VMAT) using Halcyon^TM^ or TrueBeam^TM^ linear accelerators (Varian Medical Systems, Palo Alto, CA, USA).

### 2.5. Statistical Analyses and Outcome Measures

General descriptive statistics were calculated using IBM SPSS Statistics 24 (IBM Corporation, Somers, NY, USA) and GraphPad Prism 9.1 (GraphPad Software, San Diego, CA, USA). Boxplots and further graphical representations were created with GraphPad Prism 9.1, OriginPro 2021b (OriginLab Corporation, Northampton, MA, USA). Two-sided Spearman correlation tests and the corresponding coefficients were used to compare differences in parameters between single measurements. Mann–Whitney U tests were employed to analyze non-normally distributed continuous variables, whereas *t*-tests were utilized for normally distributed variables. For nominal variables, the analysis relied on the Chi-square test and Fisher’s exact test. The statistical significance level was set to 0.05 for all analyses.

## 3. Results

### 3.1. Patient and Treatment Characteristics

In total, 132 patients were included, of whom 19 patients underwent ThuLEP and 23 patients underwent TURP before radiotherapy. The median age was 74 years for those with prior surgery and 71 years for those without (Table 1). The median prostate volume in the group with previous surgery was 18 mL before radiotherapy, significantly lower than in the group without TURP/ThuLEP (39 mL; *p* < 0.001). The PSA value before RT also differed between the groups (8.3 ng/mL with ThuLEP/TURP vs. 10.5 ng/mL without surgery, *p* = 0.08), as did the median EBRT dose administered (78.9 Gy vs. 73.8 Gy without surgery; *p* = 0.01). There was a significant difference between TURP and ThuLEP concerning the initial PSA value (8.6 ng/mL: TURP vs. 7.9 ng/mL: ThuLEP; *p* = 0.01); however, the other values were not significantly different between the groups. In all three groups, approximately 50% of patients received androgen deprivation therapy (ADT) for 9 to 12 months.

The median follow-up was 36 months.

**Table 1:** Patient characteristics compared between prior TURP and ThuLEP as well as between patients with and without prior surgery.

Parameter	TURP (n = 23)	ThuLEP (n = 19)	*p* Value	TURP or ThuLEP (n = 42)	No TURP/ThuLEP (n = 90)	*p* Value
Median age at diagnosis (years)	74 (range, 54–87)	74 (range, 66–90)	0.498	74 (range, 54–90)	73 (range, 51–89)	0.702
Median pre-treatment PSA (ng/mL)	**8.6 (0.3–92.5)**	**7.9 (0.7–32)**	**0.01**	**8.3 (0.3–92.5)**	**10.5 (range, 0.1–84)**	**0.08**
T stage: T1–2a/T2b–T2c/T3–T4/TX (%)	78/9/13/0	74/5/21/0	0.546	76/7/17/0	71/23/6/0	0.115
Median pre-RT prostate volume (cm^3^)	18 (range, 12–35)	17 (range, 10–30)	0.651	**18 (range, 10–35)**	**39 (range, 22–123)**	**<0.001**
Median pre-TURP/ThuLEP prostate volume (cm^3^)	78 (range, 34–120)	89 (range, 55–180)	0.651	80 (range, 34–180)	39 (range, 22–123)	<0.001
N stage: N0/N1/NX (%)	88/22/0	84/5/11	0.539	81/14/5	90/10/0	0.597
Gleason score: 6/7/≥8 (%)	26/39/35	16/31/53	0.159	19/36/45	13/51/36	0.859
Risk stratification: low/intermediate/high risk (%)	26/30/44	16/26/58	0.448	21/29/50	6/51/43	0.392
PIRADS-Score by mpMRI: 3/4/5 (%)	20/40/40	24/29/47	0.189	21/36/43	8/30/61	0.825
EBRT dose (Gy)	78.9 (range, 72.0–80.0)	78.8 (range, 72.0–80.0)	0.734	**78.9 (range, 72–80)**	**73.8 (range, 72–80)**	**0.013**
Radiation technique: 3D-CRT /static/dynamic IMRT (dynamic: VMAT/Tomotherapy) (%)	0/13/87	0/11/89	0.656	0/12/88	0/27/73	0.926
Median bladder volume receiving 70 Gy (V_70Gy_) (cm^3^)	31 (range, 4–73)	33 (range, 6–77)	0.859	32 (range, 4–77)	35 (range, 5–81)	0.065
Use of ADT/mean duration (%/months)	56/9	47/12	0.845	52/11	56/9	0.634
Median follow-up (months)	30 (range, 12–84)	36 (range, 18–48)	0.17	36 (range, 12–98)	36 (range, 8–60)	0.473

Significant values are printed in bold if *p* < 0.05. Mann–Whitney U tests were used for non-normally distributed continuous variables. *t*-tests were used for normally distributed variables. A Chi-square test was performed for nominal variables. Abbreviations: PSA: prostate-specific antigen; T: tumor; N: nodus/lymphonodal status; TURP: transurethral resection of the prostate; ThuLEP: laser enucleation of the prostate; PI-RADS: Prostate Imaging Reporting and Data System Guidelines; MpMRI: multiparametric magnetic resonance imaging; EBRT: external beam radiotherapy; 3D CRT: 3D conformal radiotherapy; IMRT: intensity-modulated radiotherapy; VMAT: volumetric modulated arc therapy; ADT: androgen deprivation therapy.

### 3.2. Comparison between TURP and ThuLEP: Patients with Prior TURP Showed a Significant Increase in IPSS and QoL Immediately Post-RT

**Figure 1:**
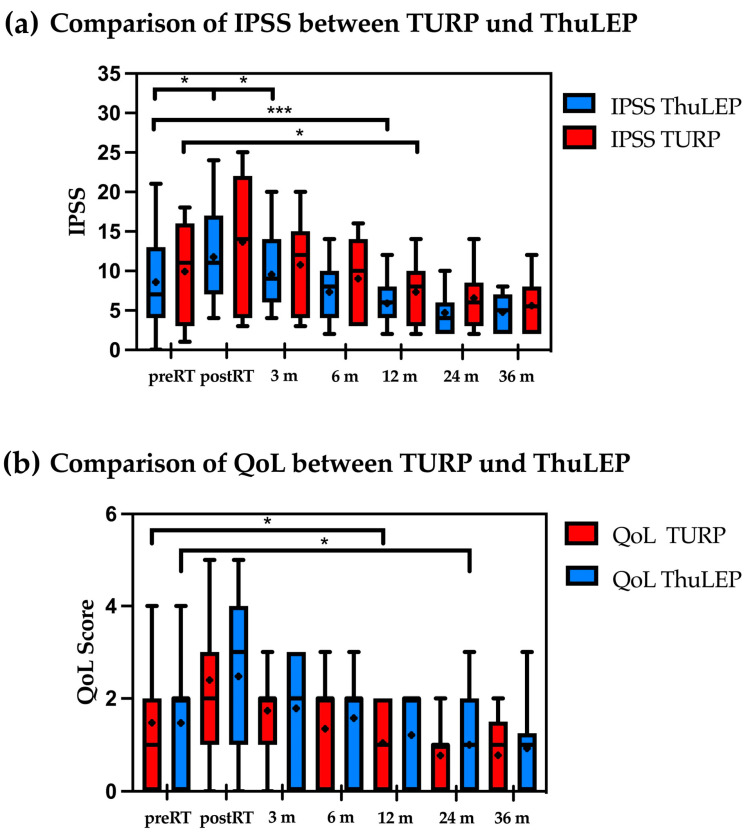
(**a**,**b**) Comparison of IPSS (**a**) and QoL-score (**b**) between patients who underwent TURP and ThuLEP before and after radiotherapy. * *p* < 0.05; ** *p* < 0.01, *** *p* < 0.001, **** *p* < 0.0001, ns = not significant. Median is marked as horizontal line and mean is marked as +. Abbreviations: IPSS: International Prostate Syndrome Score; m: months; QoL: quality of life; RT: radiotherapy; ThuLEP: thulium laser enucleation of the prostate; TURP: transurethral resection of the prostate.

Patients who underwent previous TURP showed a significant IPSS increase immediately post-RT (*p* = 0.02), while patients with ThuLEP showed a trend but no significant increase (*p* = 0.07, Figure 1a). However, after 12 months, both groups showed a decrease in the IPSS compared to the baseline (for TURP: *p* = 0.002; from mean 8.6 to 5.9; for ThuLEP patients from 9.9 to 7.3, *p* = 0.01). IPSSs remained reduced for the rest of the available follow-up. With regard to the QoL score, patients with prior TURP showed a difference after 12 months post-RT compared to the initial value before RT (*p* = 0.02).

Conversely, patients with prior ThuLEP showed a significant difference after 24 months (*p* = 0.04), indicating a longer period until the initial symptoms improved (Figure 1b).

### 3.3. Patients Who Underwent TURP/ThuLEP Showed Higher IPSS Post-RT and a Longer Period until the Baseline Value Was Reached

**Figure 2:**
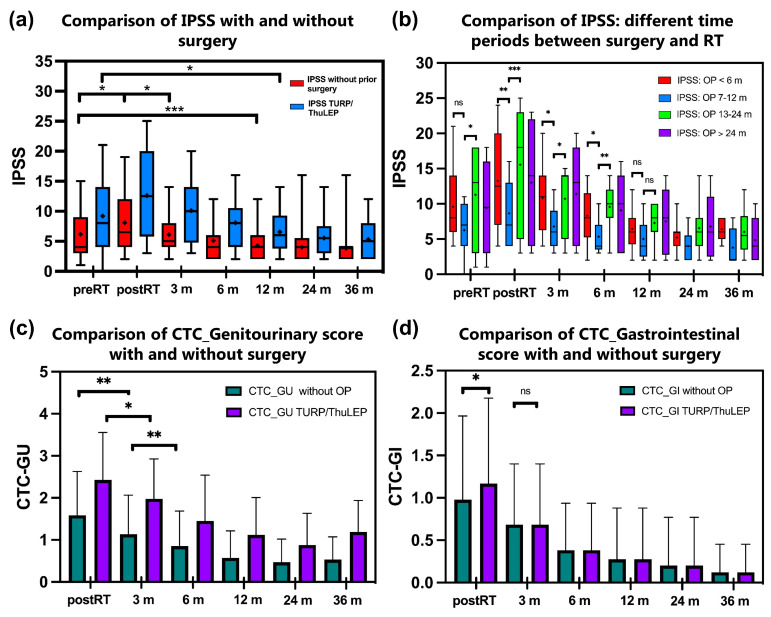
(**a**–**d**). Comparison of IPSS between patients who underwent TURP or ThuLEP before radiotherapy with patients without prior surgery (**a**). Figure 2b shows a subgroup analysis of patients who underwent surgery at different times before RT (**b**). Particularly, the subgroup of patients who underwent surgery between 6 and 12 months before radiotherapy shows a tendency of lower toxicity especially post-RT up to 6 months after RT compared to the other groups. Figure 2c,d show courses of patient-reported urinary toxicity (**c**) and gastrointestinal toxicity (**d**) measured with the Patient-Reported Outcome Common Terminology Criteria for Adverse Events. * *p* < 0.05; ** *p* < 0.01, *** *p* < 0.001, ns = not significant. The median is marked as a horizontal line and the mean is marked as +. Abbreviations: CTC-GU: Common Terminology Criteria for Adverse Events Genitourinary score; CTC-GI: Common Terminology Criteria for Adverse Events Gastrointestinal score; IPSS: International Prostate Syndrome Score; m: months; OP: operation; RT: radiotherapy; ThuLEP: thulium laser enucleation of the prostate; TURP: transurethral resection of the prostate.

Patients without previous surgery show a significant increase in the IPSS directly after RT (*p* < 0.001, Figure 2a). However, baseline values were reached again after 3 months, and the IPSS is lower compared to the baseline value after 6 months (*p* < 0.001). For patients with previous TURP/ThuLEP, the IPSS is significantly higher immediately after RT (*p* = 0.004). It takes 6 months longer for the IPSS to be lower than the baseline in this group (difference between baseline IPSS and IPSS after 12 months: *p* < 0.001). The QoL score has similar values in both groups before RT (cf. 1.27 vs. 1.48). However, there is a significant increase in the QoL score in the group with previous OP compared to the patients without previous TURP/ThuLEP.

The CTC-GU score also shows a significant difference between the two groups with higher values for patients with previous TURP/ThuLEP. Regarding the CTC GI score, there is a similar trend but no significant difference (Figure 2c,d). Further differences between the groups are shown in Supplementary Table S2.

### 3.4. Comparison of Different Time Periods between Surgery and Radiotherapy Shows That a Period between 6 and 12 Months Is Associated with Less Toxicity

In a further subgroup analysis, the patients were categorized according to different periods between surgery and RT (Figure 2b). Patients with RT > 12 months after surgery showed tendencies of higher IPSS values and thus higher toxicity without clear significance. Patients with surgery between 7 and 12 months before RT showed a tendency towards significantly lower IPSS values especially in the course post-RT (_IPSSmean7–12months_ 5.4 ± 3.3 vs. IPSS_mean < 6months_ 10.2 ± 5.3, *p* = 0.002, and vs. IPSS_mean13–24months_ 11.3 ± 6.8, *p* = 0.01) albeit with the limitation of a small total number of cases (n = 42).

### 3.5. No Significant Differences in a Further Small Subgroup Analysis of Patients with Different Prostate Volumes before TURP/ThuLEP and before Radiotherapy

In a subgroup analysis, patients without previous surgery were compared in terms of different prostate volumes before RT (Supplementary Figure S1). Patients with volumes below 50 mL, around 50–80 mL, and above 80 mL showed similar toxicities after RT. Patients with previous surgery showed increased toxicities immediately post-RT and especially 3 months after RT (for operated patients: IPSS_3monthsAfterRT_ 10.1 ± 5.2 vs., e.g., for patients without surgery with initial prostate volume <50 mL: IPSS_3monthsAfterRT_ 6.0 ± 3.4, *p* < 0.001).

Further multivariate analyses demonstrate correlations between heart disease and elevated scores in the group of patients who had not undergone surgery. Concomitant ADT also correlates with increased score values and thus with increased urinary symptom burden. Achieving a total dose above 79.0 Gy correlated with increased IPSS values only in the group of patients with TURP/ThuLEP (see Table 2). Increased age and higher T stages showed no further significant differences between the groups in the analysis.

## 4. Discussion

In this study, we analyzed urinary and intestinal toxicity profiles in acute and late settings in patients who received EBRT for localized prostate cancer with and without previous TURP or ThuLEP. Changes in urinary functions affecting the post-treatment QoL were additionally evaluated.

### 4.1. GU Toxicities in Patients with Prior TURP Versus ThuLEP and Versus Non-Operated Patients

Previously, a superior urinary functional outcome with a lower postoperative toxicity profile was demonstrated in patients after laser enucleation of the prostate compared to the TURP procedure [2,6,21,22]. In accordance, the development of GU toxicities after EBRT was found more favorable after ThuLEP in this study (Figure 1a,b). After 12 months, the urinary symptoms normalized in both groups. Interestingly, urinary function in both therapy groups was improved beyond baseline measurements 24 months after EBRT, as demonstrated by reduced IPSSs. In contrast to Devisetty et al. [22], our study revealed the association of grade 3 GU toxicity only with urinary incontinence and not with hematuria. Devisetty et al. analyzed 71 patients with prior TURP before EBRT in 2007. They observed that late grade 3 or higher GU toxicity was more common in patients with TURP compared to those without TURP (16% vs. 4%). The risk ratio for a late grade 3 or higher GU toxicity after TURP was 2.87. In this study, only 31% of the patients were irradiated using the IMRT technique which might be one explanation for the different results. Because the extent of periurethral fibrosis might be the key determinant for inducing postoperative immediate and long-term side effects, different surgical techniques over time are also possible explanations for the difference. Our results correlate with most of the surgical literature, where the grade 3 events were related to incontinence in acute and late settings [6,23,24]. The prevalence of incontinence suggests that the EBRT toxicities develop within the urethral sphincter because of fibrosis after surgery at the level of the bladder neck sphincter [10,22]. The extent of fibrotic changes after TURP might be more extensive than after ThuLEP, leading to a higher rate of GU side effects after EBRT in TURP patients.

For EBRT, there is a wide array of evidence regarding the influence of prior TURP on GU toxicity. Most published studies suggest that TURP is one of the risk factors for GU toxicity during and after RT [7,9,11]. We did not analyze patients undergoing surgery after RT as shown in various prior investigations [25,26]. A series of evidence showed that TURP before EBRT increases the risk of overall GU toxicity [27,28], urethral strictures [10,29], and urinary incontinence [7,30,31].

In contrast to TURP, there is no evidence of the effects of ThuLEP on GU toxicity when RT is performed after this procedure. With both Holmium Laser Enucleation of the Prostate (HoLEP) and ThuLEP employing a similar laser enucleation procedure, Laughlin et al. found no significant rise in urinary incontinence post-radiation therapy (RT) compared to pre-RT levels in their analysis of 18 patients who had undergone HoLEP [32]. The median IPSS after a median follow-up of 13.5 months post-RT remained similar to the median IPSS before. Bladder control as well as overall GU toxicity in patients with previous HoLEP did not worsen significantly after RT. A similar GU toxicity profile and development of GU side effects in post-RT acute and long-term follow-up were obtained in patients with prior ThuLEP analyzed in this study.

GU toxicity regression took significantly longer in the previously operated group compared to non-operated patients (9.17 months vs. 6.14 months, *p* = 0.03, Figure 2c). In general, the values of the CTC urinary score decreased continuously in the post-RT time until the end of the observation period with generally higher score values in the surgery group (Figure 2c). In our analysis, patients who underwent ThuLEP had lower postoperative toxicities compared to TURP. Nevertheless, we demonstrate a relevant worsening of urinary symptoms after EBRT by comparing a total group of operated patients and non-operated patients. Thus, a significantly higher IPSS increase in operated patients compared to the non-surgery group was observed after EBRT (Figure 2a).

### 4.2. Rectal Toxicities

As shown in Figure 2d, the CTC gastrointestinal score is the only one of the scores examined that shows no significant differences between patients without and with prior surgery. The values decrease continuously over time after completing definitive RT. Pinkawa et al. found larger acute bowel score changes and significantly slower recovery in patients without prior TURP compared to patients with prior TURP [33]. Laughlin et al. found maximum grade 1 acute and late rectal toxicity in patients with a history of HoLEP [32]. These results correlate with our evidence where the manifestation grade and rate of rectal symptoms after RT were very low in patients with prior prostate surgery. In general, a reduced prostate volume after subtotal resection can be a relevant factor for optimized protection of the rectum by EBRT of the prostate. Conversely, non-operated patients are anticipated to experience increased radiation exposure to the rectum, attributed to the proximity of the prostate to this organ. This might be an explanation for our observation of a correlation between larger prostate volumes and a higher IPSS only in the group without prior surgery.

### 4.3. Quality of Life in Relation to GU Toxicities

The analysis of the QoL score delivers similar results as the IPSS measure. Patients who underwent TURP showed significantly lower QoL values compared to baseline after 12 months, while this only occurred after 24 months in patients with prior ThuLEP (Figure 1b). Besides the QoL score, the “Expanded prostate cancer index composite” (EPIC) score to assess the QoL outcomes in the RT of prostate cancer is often used in the literature [33,34,35,36]. Using the EPIC QoL score, Pinkawa et al. found significant differences in urinary changes at the end of EBRT between patients with versus without prior TURP [33]. According to our results, no significant QoL differences for urinary toxicities were found in a long-term follow-up (36 months post-RT). In our data, radiation dose was a risk factor for higher IPSS and QoL values in the operated group (Table 2).

### 4.4. Parameters Affecting Toxicity Profile

#### 4.4.1. Use of ADT

The multivariate analysis in our study demonstrates the association between the use of ADT and increased IPSS in acute and late settings. This result is in accordance with data received by Devisetty et al., who found a higher incidence of late GU toxicity after EBRT in TURP patients with concomitant ADT [22]. In contrast, Zapatero et al. suggested ADT as a protective factor for the development of late GU toxicities [37,38]. We hypothesize that periurethral fibrosis is a key factor for the development of late urinary toxicities after EBRT in operated patients, where the effect of ADT on fibrotic tissue is maximally reduced. On the contrary, in non-operated patients, the use of ADT can potentially significantly reduce the manifestation of GU and GI side effects by downsizing the prostate with a better possibility of rectum protection in radiation treatment plans.

#### 4.4.2. Radiation Treatment Technique

The analyzed patients in this study were treated by using the dynamic IMRT technique. Comparing the incidence and rate of toxicities after EBRT in operated patients, we found a significantly lower rate of acute grade 2 and higher GU toxicities than in other studies, where the patients were treated using older radiation techniques including 2D-CRT, 3D-CRT, or static IMRT [7,28,29,39]. Regarding the late toxicities, we did not find any increased rate of urinary incontinence, obstructive symptoms, or hematuria at 4 years compared to baseline values. In contrast, Sandhu et al. found a significantly higher rate of urethral strictures after 5 years by using 3D-CRT and static IMRT treatment techniques analyzing 120 TURP patients [29]. Similarly, Liu et al. revealed an increased rate of grade 1 urinary incontinence or higher at 5 years by analyzing 246 TURP patients who were treated with 2D-CRT or 3D-CRT [7]. It is conceivable that an improved GU toxicity profile in acute and late settings in our study was achieved by using dynamic IMRT in most patients that deliver high conformity dose distribution with maximal urinary bladder and rectum protection. Secondly, approximately half of the analyzed patients received ThuLEP which might explain the lower rate of periurethral and bladder neck fibrosis compared to the TURP procedure [9,21,40,41].

#### 4.4.3. The Time Interval between Surgery and EBRT

We found a tendency towards a significant decrease in acute urinary toxicity (up to 3 months after EBRT) in patients who were irradiated between 7 and 12 months after surgery, especially in contrast to patients with an interval over 12 months between surgery and EBRT (Figure 2b, *p* = 0.01). This result indicates that a longer time for EBRT might be a risk factor for urinary toxicity. Due to the small number of patients in the different groups (n_overall_ = 42), further analyses are necessary for further conclusions regarding an optimal time course between surgery and RT. Pinkawa et al. discovered that the duration of late toxicity, such as urinary incontinence, was prolonged for patients with a greater interval between resection and EBRT [33]. Finally, Devisetty et al. did not find any significant impact of the time interval between surgery and EBRT in the development of acute and late GU side effects [22]. Considering a multifactorial impact on the development of radiation-induced side effects in operated patients, the effect of time intervals cannot be precisely defined yet.

### 4.5. Limitations

Like all retrospective cohort studies, our results may be biased due to factors such as patient selection, the extended study duration, the absence of control for TURP and ThuLEP procedures, and incomplete clinical data. A more comprehensive dataset, including details regarding the specific type of TURP or ThuLEP procedure and the volume of prostate tissue removed, may have identified additional potential prognostic factors. Case numbers were limited with regard to patients who underwent TURP and ThuLEP (n = 42) and so the small subgroup analyses indicate a trend that still needs to be proved in larger studies.

### 4.6. Clinical Prospects

The currently increased use of ThuLEP and HoLEP has not yet been prospectively investigated regarding radiotherapy-associated side effects. The results of our study showed that after 36 months post-RT, there is generally no significant difference between the toxicities of operated and non-operated patients. It is therefore worthwhile for a prospective study design to take a closer look at the toxicities before and up to 2 years after radiotherapy. The timing of the previous surgical treatment is important with regard to acute and early late toxicities: a period of time that is too short can be unfavorable due to acute side effects and a period of time that is too long can be unfavorable due to fibrotic changes (see Pinkawa et al. [33]), so it is clinically relevant to determine whether the period of 7–12 months between surgery and radiotherapy indicated in our study proves to be the most favorable. Other important parameters influencing toxicities and restrictions in quality of life around radiotherapy are the number of operations, as many patients also undergo TURP or ThuLEP several times, as well as the initial volume and the remaining volume of the prostate after surgery. The impact of different surgical techniques (TURP and ThuLEP) and of different prostate volumes diverge in the literature. The same applies to the simultaneous use of ADT in pre-operated patients with TURP/ThuLEP who receive definitive radiotherapy. Further prospective analyses may provide clinically relevant information and identify risk factors for toxicities before radiotherapy is started so that intervention measures can be initiated at an early stage. Moreover, adaptive radiotherapy offers the possibility of better protection of the organs at risk, particularly in patients with pre-existing risk factors such as a previous TURP and ThuLEP. It may be especially useful to identify patients with increased micturition and defecation scores before radiotherapy as a vulnerable patient group for adaptive planning. These groups can be compared with conventional procedures in order to determine optimal radiation treatment.

## 5. Conclusions

Definitive RT to the prostate in patients with a history of TURP or ThuLEP shows increased toxicity in the short term compared to non-operated patients, but effects largely dissipate 24 months after RT. Intriguingly, symptoms improve beyond pre-RT baseline values in the longer term in these patients despite the application of high-dose EBRT in the interim. Patients with prior ThuLEP show a more favorable urinary toxicity profile in the first 12 months after EBRT than TURP patients. EBRT is an appropriate treatment option for localized prostate cancer in patients with a history of TURP and ThuLEP, but short-term symptom worsening should be anticipated. In sum, we suggest that the elevated risk of late toxicities may be especially linked to different radiation techniques, doses, and the time between operation and RT, as well as the degree of surgery-induced fibrosis within the radiation field. Moreover, this fibrosis might increase over time following the surgery.

## Figures and Tables

**Table 2:** Factors associated and compared with IPSS 8 or higher and grade 3 GU toxicities or higher evaluated in multivariate analysis.

Factor	*p* Value Patients with Prior ThuLEP and TURP (IPSS preRT/Immediately Post-RT)	*p* Value Patients without Surgery (IPSS preRT/Immediately Post-RT)
Age at diagnosis (years): ≥80	0.477/0.972	0.499/0.672
T stage: ≥T2c	0.097/0.298	0.308/0.807
External beam radiotherapy (EBRT) dose (Gy): ≥79.2	**0.047**	0.259
Radiation technique: volumetric intensity arc therapy (VMAT instead of static IMRT)	0.434	0.61
Use of androgen deprivation therapy (ADT)	**0.006/<0.001**	**0.032/0.003**

Significant values are printed in bold if *p* < 0.05. Mann–Whitney U tests were used for non-normally distributed continuous variables. *t*-tests were used for normally distributed variables. A Chi-square test was performed for nominal variables.

## Data Availability

The data that support the findings of this study are available from the corresponding author upon reasonable request.

## Supplementary Materials

The following supporting information can be downloaded at https://www.mdpi.com/article/10.3390/cancers16193403/s1: Supplementary Figure S1: A subgroup analysis of patients with different prostate volumes before RT without prior surgery; Supplementary Table S1: Inclusion and exclusion criteria of the study; a retrospective analysis of 132 patients who underwent radiotherapy between 2012 and 2021 (42 with prior surgery, 90 without prior surgery); Supplementary Table S2: Toxicity scores divided into severity levels with percentages of patients and associated significance between patients who underwent surgery and those without TURP/ThuLEP.
