# Supplementary material for: Effect of Prior Transurethral Prostate Resection (TURP) or Laser Enucleation (ThuLEP) on Radiotherapy-Induced Toxicity and Quality of Life in Prostate Cancer Patients Undergoing Definitive Radiotherapy
Source: Cancers (Basel). 2024 Oct 6;16(19):3403. doi: 10.3390/cancers16193403 (PMC11476121; doi:10.3390/cancers16193403)
Supplement: Supplementary file 1 [file cancers-16-03403-s001.zip › Supplementary Figure S1.pdf]

Supplementary Figure S1. Comparison of IPSS with different prostate volumes of patients without and with surgery

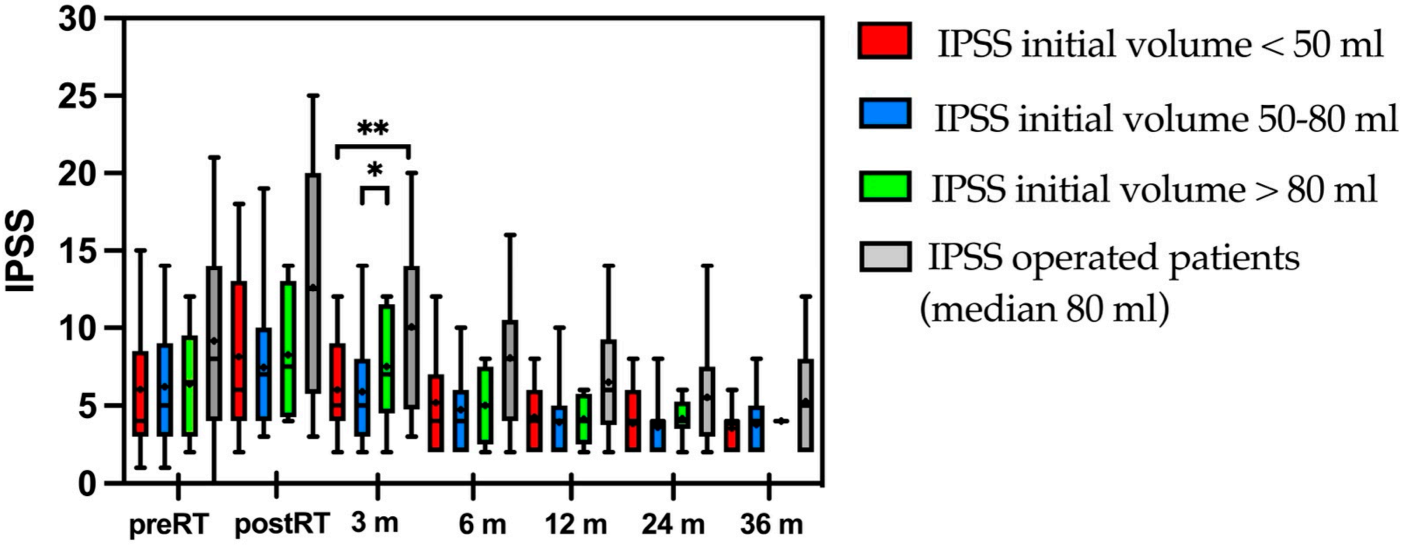

A subgroup analysis of patients with different prostate volumes before RT without prior surgery. The groups are also compared with patients who underwent surgery with a median prostate volume of 80 ml (before RT). Overall, the number of patients per group is small, so that only a limited state-ment is possible. Patients with a V > 80 ml show a tendency for stronger side effects of RT without direct significance. Operated patients show significantly higher IPSS values, especially immedi-ately postRT and 3 months postRT. * p < 0.05; ** p < 0.01, *** p < 0.001, **** p < 0.0001, ns = not significant. Median is marked as horizontal line and mean is marked as +.

Abbreviations: IPSS: International Prostate Syndrome Score; m: months; RT: Radiotherapy.
