# Supplementary material for: Effect of Prior Transurethral Prostate Resection (TURP) or Laser Enucleation (ThuLEP) on Radiotherapy-Induced Toxicity and Quality of Life in Prostate Cancer Patients Undergoing Definitive Radiotherapy
Source: Cancers (Basel). 2024 Oct 6;16(19):3403. doi: 10.3390/cancers16193403 (PMC11476121; doi:10.3390/cancers16193403)
Supplement: Supplementary file 1 [file cancers-16-03403-s001.zip › Supplementary_Table_S1.pdf]

Supplementary Table S1. Inclusion and exclusion criteria of the study; a retrospective analysis of 132 patients who underwent radiotherapy between 2012 and 2021 (42 with prior surgery, 90 without prior surgery).

Inclusion criteria	Age ≥ 50
	Intensity modulated radiotherapy (IMRT) technique for oncologic treatment
	Diagnosis of prostate cancer: a) primary localised / locally advanced prostate cancer with or without lymphonodal pelvin metastases; b) prostate cancer with osseous oligometastasis (up to 5 metastases)
	Radiation dose: according to guidelines of the European Association of Urology (EAU) updated in 05/2023
	Registered acute and late genitourinary side effects; radiation treatment plans with dose-volume histograms must have been available for analysis
Exclusion criteria	Pre-treatment such as radiotherapy or systemic therapy (chemotherapy/immunotherapy) before surgery (TURP or ThuLEP)
	Comorbidities that severely impair the general condition such as a metastatic second tumor
	To describe the patient's general condition the performance status according to the Eastern Cooperative Oncology Group (ECOG) was used. Patients with ECOG ≥ 3 before radiotherapy were excluded
