# Supplementary material for: Effect of Prior Transurethral Prostate Resection (TURP) or Laser Enucleation (ThuLEP) on Radiotherapy-Induced Toxicity and Quality of Life in Prostate Cancer Patients Undergoing Definitive Radiotherapy
Source: Cancers (Basel). 2024 Oct 6;16(19):3403. doi: 10.3390/cancers16193403 (PMC11476121; doi:10.3390/cancers16193403)
Supplement: Supplementary file 1 [file cancers-16-03403-s001.zip › Supplementary_Table_S2.pdf]

Supplementary Table S2. Toxicity scores divided into severity levels with percentages of patients and associated significance between patients who underwent surgery and those without TURP/ThuLEP.

**Toxicity score**	**Time point**	**TURP/TULEP (n=42)**	**No TURP/TULEP (n=90)**	**p value (differences in IPSS between patients with and without surgery)***
IPSS-Score, Low (<8), Intermediate (8-19) and high ≥ 20; (%)	Pre-RT	19/79/2	68/32/0	**0.004**
	Post-RT	40/31/29	54/46/0	**0.001**
	3 mon.	38/57/5	64/36/0	**< 0.001**
	6 mon.	40/60/0	77/23/0	**< 0.001**
	12 mon.	55/45/0	90/10/0 (n=89)	**< 0.001**
	24 mon.	75/25/0 (n=40)	93/7/0 (n=76)	**0.006**
	36 mon.	74/26/0 (n=23)	98/2/0 (n=56)	**0.019**
Genitourinary QoL score, grade 2 or higher; (%)	Pre-RT	52	32	0.132
	Post-RT	69	40	**< 0.001**
	3 mon.	64	36	**< 0.001**
	6 mon.	64	20	**0.001**
	12 mon.	43	11 (n=89)	**0.001**
	24 mon.	19 (n=40)	8 (n=76)	0.728
	36 mon.	22 (n=23)	4 (n=56)	**0.012**
CTCAE_V5 GU Scale, grade 2 or higher; (%)	Post-RT	71	46 (n=89)	**< 0.001**
	3 mon.	64	37 (n=89)	**< 0.001**
	6 mon.	57	24 (n=89)	0.247
	12 mon.	45	8 (n=86)	**0.002**
	24 mon.	23 (n=40)	3 (n=76)	**0.01**
	36 mon.	32 (n=19)	4 (n=56)	**0.005**
CTCAE_V5 GI Scale, grade 2 or higher; (%)	Post-RT	40	31 (n=89)	0.325
	3 mon.	19	15 (n=89)	0.381
	6 mon.	12	3 (n=89)	0.159
	12 mon.	2	2 (n=87)	0.256
	24 mon.	2 (n=41)	1 (n=76)	0.313
	36 mon.	6 (n=16)	2 (n=49)	0.123

Abbreviations: **IPSS**: International Prostate Syndrome Score; **CTCAE_V5 GU/GI Scale**: Common Terminology Criteria for Adverse Events version 5 genitourinary/gastrointestinal scale
